# Human drug efflux transporter ABCC5 confers acquired resistance to pemetrexed in breast cancer

**DOI:** 10.1186/s12935-021-01842-x

**Published:** 2021-02-25

**Authors:** Jihui Chen, Zhipeng Wang, Shouhong Gao, Kejin Wu, Fang Bai, Qiqiang Zhang, Hongyu Wang, Qin Ye, Fengjing Xu, Hong Sun, Yunshu Lu, Yan Liu

**Affiliations:** 1 Department of Pharmacy, Xin Hua Hospital, Shanghai Jiao Tong University School of Medicine, 1665 Kongjiang Road, Shanghai, 200092 China; 2 Department of Pharmacy, Changzheng Hospital, Second Military Medical University, Shanghai, 200003 China; 3 Department of Breast Surgery, Obstetrics and Gynecology Hospital, Fudan University, Shanghai, 200011 China; 4 Department of Pharmacy, Provincial Clinical College of Fujian Medical University, Fujian Provincial Hospital, Fuzhou, 350001 China

**Keywords:** ABC transporter, Breast cancer, Pemetrexed, Drug resistance, Sensitivity

## Abstract

**Aim:**

Pemetrexed, a new generation antifolate drug, has been approved for the treatment of locally advanced or metastatic breast cancer. However, factors affecting its efficacy and resistance have not been fully elucidated yet. ATP-binding cassette (ABC) transporters are predictors of prognosis as well as of adverse effects of several xenobiotics. This study was designed to explore whether ABC transporters affect pemetrexed resistance and can contribute to the optimization of breast cancer treatment regimen.

**Methods:**

First, we measured the expression levels of ABC transporter family members in cell lines. Subsequently, we assessed the potential role of ABC transporters in conferring resistance to pemetrexed in primary breast cancer cells isolated from 34 breast cancer patients and the role of ABCC5 in mediating pemetrexed transport and apoptotic pathways in MCF-7 cells. Finally, the influence of ABCC5 expression on the therapeutic effect of pemetrexed was evaluated in an in vivo xenograft mouse model of breast cancer.

**Results:**

The expression levels of ABCC2, ABCC4, ABCC5, and ABCG2 significantly increased in the pan-resistant cell line, and the ABCC5 level in the MCF-7-ADR cell line was 5.21 times higher than that in the control group. ABCC5 expression was inversely correlated with pemetrexed sensitivity (IC_50_, r = 0.741; *p* < 0.001) in breast cancer cells derived from 34 patients. Furthermore, we found that the expression level of ABCC5 influenced the efflux and cytotoxicity of pemetrexed in MCF-7 cells, with IC_50_ values of 0.06 and 0.20 μg/mL in ABCC5 knockout and over-expression cells, respectively. In the in vivo study, we observed that ABCC5 affected the sensitivity of pemetrexed in breast tumor-bearing mice, and the tumor volume was much larger in the ABCC5-overexpressing group than in the control group when compared with their own initial volumes (2.7-fold vs. 1.3-fold).

**Conclusions:**

Our results indicated that ABCC5 expression was associated with pemetrexed resistance in vitro and in vivo, and it may serve as a target or biomarker for the optimization of pemetrexed regimen in breast cancer treatment.

**Supplementary Information:**

The online version contains supplementary material available at 10.1186/s12935-021-01842-x.

## Introduction

Pemetrexed (MTA), a novel multitargeted antifolate, is used for the treatment of non-small cell lung cancer and mesothelioma [1–4], and functions by inhibiting thymidylate synthase, dihydrofolate reductase, glycinamide ribonucleotide formyltransferase, and 5-aminoimidazole-4-carboxamide ribonucleotide formyltransferase, which are folate-dependent enzymes involved in the de novo biosynthesis of thymidine and purine nucleotides [5, 6]. MTA exhibits a good effect (overall response rate: 8%, stable disease state: 36%, median survival: 8 months) in metastatic breast cancer (BC) patients, and is well-tolerated in 80% of the patients receiving second-line treatment [7]. Additionally, in advanced BC patients, a 30% response rate is observed when MTA is administered as first-line treatment, whereas a 21% response rate is observed when MTA is administered as second-line treatment [8].

The mechanism of sensitivity and/or acquired resistance of MTA is complex, mainly including a decreased intracellular concentration and alteration of metabolism, among others [9]. The cytotoxicity of MTA is largely attributed to its concentration and retention time in cells [10]. Thus, the transmembrane transport of MTA is a critical determinant of its activity. In this context, a decreased expression of influx transporters and increased expression of efflux transporters can induce cancer cell resistance by reducing intracellular drug exposure [11, 12]. The ATP-binding cassette (ABC) transporter, also known as multidrug resistance protein [13], is capable of conferring resistance to nucleotide analogs such as fluorouracil (5-FU) [14, 15], 6-thioguanine (6-TG) [16], 6-mercaptopurine (6-MP) [17], and 9-(2-phosphonylmethoxynyl)adenine [18, 19]. The expression of ABC transporters involved in the efflux of endogenous and exogenous substrates is regulated by pregnane X receptor, constitutive androstane receptor, and other transcriptional regulators [20]. ABC transporters comprise approximately 50 members, which are subdivided into seven groups (ABCA to ABCG) [21]. In particular, the C branch of ABC transporter superfamily dominates multidrug resistance [22, 23]. The first study on hydrophilic antifolate transport by certain members of the ABCC family reports that MTA transports into bile in wild-type rats but not in rats with a hereditary deficiency of ABCC2 functions [24]. Subsequently, many studies have reported the effects of ABCC on antifolate transport [25–28]. An absence of ABCC2 and/or ABCG2 in mice increases the oral availability of methotrexate [29]. Another important exporter, ABCC5, is reported to be involved in the transport of MTA and folic acid in HEK293 cells [30].

Previous studies have shown that the expression of ABC transporters in human cancer can influence the efficacy of chemotherapy. However, the contribution of ABC transporters to drug resistance, especially to novel antifolate drugs, has not been fully elucidated. Therefore, this study was conducted to explore the influence of ABC transporters on MTA sensitivity and resistance in BC.

## Materials and methods

### Cell lines

Breast cancer cell lines, MCF-7 and MCF-7-adriamycin-resistant cells (MCF-7-ADR), were obtained from the Culture Collection Company (ATCC-LGC Promochem, Teddington, UK). Cells were routinely grown in Dulbecco's modified eagle's medium (DMEM, Invitrogen, Carlsbad) supplemented with 10% fetal bovine serum (FBS, Invitrogen) and 100 units of penicillin/streptomycin per mL (Invitrogen) at 37 °C, and they were kept in a humidified environment under 5% CO_2_. The medium was replaced regularly every 2–3 days until the cells reached 80–90% confluence. Following this, the cells were transferred to perform experiments or make stock solutions. Mycoplasma contamination was tested every month.

### Reagents

MTA (reference substance solution), which was used for quantitative analysis in a reverse phase-high performance liquid chromatography (RP-HPLC) experiment, was purchased from Sigma-Aldrich (St. Louis, MO, USA). MTA used for cell incubation was purchased from Eli Lilly Company (Indianapolis,USA). ABCC5 human shRNA was purchased from OriGene (Locus ID: 10,057, Product ID: TL315024, Rockwell, USA). Rabbit anti-human anti-MRP5 antibody [M5II-54, ab137070, Abcam, Cambridge, UK), rabbit anti-human Bax mAb (5023, Cell Signaling Technology, Boston, USA), rabbit anti-human caspase-3 mAb (14,220, Cell Signaling Technology), cleaved caspase-3 mAb (9664, Cell Signaling Technology, Boston, USA), and β-actin (13E5) rabbit mAb (4970, Cell Signaling Technology, Boston, USA) were used as primary antibodies, and goat anti-rabbit IgG (final dilution 1:2000; LI-COR Biosciences, Lincoln, NE, USA) was used as a secondary antibody.

### Construction of ABCC5 adenovirus

For recombinant adenovirus construction, ABCC5 cDNA (AdvABCC5) and green fluorescent protein cDNA (AdvCtrl, control) were cloned using PCR, and were inserted into the pHBAD-EF1-MCS-3flag-CMV-EGFP vector (Additional file 1: Fig. S1). pDC315-ABCC5 and pBHGloxE1,3Cre were co-transfected into HEK293 cells (ATCC, Manassas, USA) using the LipoFiterTM transfection reagent (QIAGEN, Dusseldorf, Germany) to generate recombinant adenoviruses. The recombinant adenoviruses, AdvABCC5 and AdvCtrl, produced in HEK293 cells were purified, and viral titer was measured using a plaque assay. The stock solutions of both AdvABCC5 and AdvCtrl contained 1 × 10^11^ plaque formation unit (PFU)/ mL.

### Collection of tumor tissue specimens and isolation of primary BC cells

A total of 34 patients with confirmed primary BC (2 cm or larger), who consecutively underwent neoadjuvant chemotherapy with anthracyclines at the Breast Tumor Department, Xin Hua Hospital, Shanghai Jiao Tong University School of Medicine, were enrolled in the study from January 2014 to December 2015. Tumor specimens were surgically excised prior to MTA chemotherapy. Informed consent was obtained from all patients, following a protocol approved by the Ethics Committee of Xin Hua Hospital, Shanghai Jiao Tong University School of Medicine.

To isolate BC cells, at least two consecutive frozen sections were prepared for each paraffin-embedded tumor tissue sample, and one of the sections was subjected to hematoxylin–eosin staining to confirm the presence of cancer cells. The adjacent sections were transferred for cancer cell isolation, as previously described [30]. Briefly, the blood, and fat and fibro connective tissue were removed from tumor tissue samples. Subsequently, the residual section was cut into 1–2 mm pieces for enzymatic disaggregation. The small tissue pieces were incubated with 2.5% crude trypsin (QIAGEN, Dusseldorf, Germany) for 30 min at 37 °C and 0.15% collagenase (QIAGEN, Dusseldorf, Germany) overnight. Cells released after enzymatic treatments were further tested for cell viability, and they were cultured to perform subsequent experiments.

### Animal studies

Twenty-four female BALB/c nude mice (5 weeks, 18 g) were purchased from the Shanghai Super B&K Laboratory Animal Corp. Ltd. (Shanghai, China), and they were raised in a specific pathogen-free environment with free access to food and water. All animal studies were approved by the Research Ethics Committee of Xin Hua Hospital, affiliated to the Shanghai Jiao Tong University School of Medicine.

**Fig. 1:**
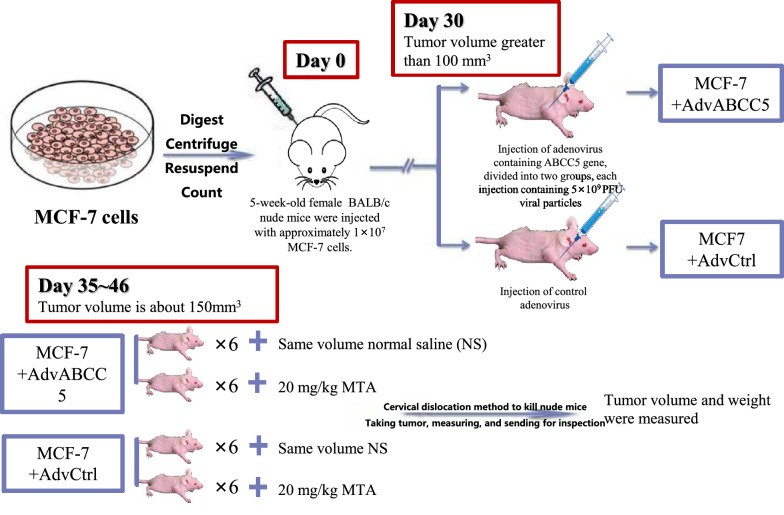
Schematic diagram of animal study to evaluate the influence of ABCC5 expression on pemetrexed (MTA) efficacy

On Day 0, 1 × 10^7^ MCF-7 cells were subcutaneously injected into the right armpits of mice. After the development of tumors to approximately 100 mm^3^ on Day 30, adenoviruses containing ABCC5 (AdvABCC5, 5 × 10^11^ PFU) were injected into the tumors of 12 mice to overexpress ABCC5 in tumor cells, and the vehicle AdvCtrl was applied to the other 12 control mice. The expression of ABCC5 was checked using diffuse green fluorescence. When the volume of tumors was approximately 150 mm^3^ on Day 35, six of the ABCC5-overexpressing mice and six control mice were intravenously injected (via the tail vein) with MTA (20 mg/kg, saline) once a day, and the same dose of vehicle was administered to the others from Day 35 to 46. The tumor volumes (V) were measured using a caliper once a day (V = width^2^ × length/2). Mice were killed at the end of seventh week, and tumor volume and weight were measured. The design of animal experiments is shown in Fig. 1.

### Determination of MTA in MCF-7 cells using RP-HPLC

MTA concentrations in MCF-7 cells were determined based on a developed HPLC method. Chromatographic separation and quantification were performed using an RP column (ZORBAX Eclipse XDB-C8, 250 mm × 4.6 mm, 5 μm; Agilent, Santa Clara, USA) with the column temperature maintained at 25 °C. MTA was detected using an ultraviolet detector (Agilent 1260 HPLC, Agilent, Santa Clara, USA) at a wavelength of 240 nm. The mobile phase was composed of water and 0.02 M phosphate buffer (pH 4.0)/acetonitrile (86:14, V:V), and it was delivered at a flow rate of 1 mL/min. Sample pretreatment was completed using ultrafiltration (0.22 μm). All experiments were performed using an Agilent 1260 HPLC system. A calibration curve was constructed in the range of 80–625 ng/mL for MTA measurement. The injection volume was 20 μL, and all experiments were performed in triplicate.

### Cell preparation for cellular uptake analysis

MCF-7 cells were seeded at 2 × 10^5^/well into six-well flat-bottom tissue-culture plates in triplicate. After 24 h, the cells were infected with ABCC5-, Ctrl-, and siABCC5-RNA-expressing adenoviruses. The cells were incubated with adenoviral particles for another 24 h; subsequently, they were refreshed with a medium containing 50 μM MTA (21.37 μg/mL). At specific time points (0, 0.5, 1, 2, 4, and 24 h), the cells were washed three times with cold phosphate buffered saline (PBS, 0.1 M, pH 7.4, Sigma-Aldrich, St. Louis, USA), resuspended in 0.2 mL RPMI-1640 medium (Sigma-Aldrich, St. Louis, USA), and homogenized. After centrifugation at 13,000 × g for 10 min, the supernatant was harvested and stored at − 80 °C for the detection of MTA using RP-HPLC, as mentioned above.

### Cell viability assay

The cell viability assay was performed using a CCK-8 kit (Dojindo Laboratories, Kumamoto, Japan) according to manufacturer's instructions. Briefly, cells were plated in 96-well plates at a density of 2000 cells/well, and subsequently, they were treated with different concentrations of MTA for 72 h. Following this, CCK-8 solution diluted with DMEM/F12 and 10% FBS at a 1:10 ratio was added to each well, and the plate was incubated at 37 °C for 2 h. Finally, absorbance was measured using a SYNERGY microplate reader (BioTek, Winooski, USA) at 450 nm. The (%) cell viability was calculated using the following formula: (OD treatment group-OD blank)/(OD control group-OD blank) × 100. IC_50_ values were determined using the GraphPad Prism software (GraphPad Prism software, Inc. San Diego, USA). All experiments were performed in triplicate, and the presented data represent the mean of three biological repeats.

### Western blotting

Tissues and cells were homogenized in ice-cold 1 × RIPA lysis buffer (Thermo Fisher Scientific, Waltham, USA) containing protease inhibitor cocktail (Roche, Basel, Switzerland), and they were centrifuged at 12,000 × g for 15 min. Protein extracts were separated using 5 to 12% SDS-PAGE (Beyotime Biotechnology, Shanghai, China), and subsequently, they were electrophoretically transferred to nitrocellulose membranes (Bio-Rad, R&D systems, Emeryville, USA). After blocking with 5% fat-free milk, the membranes were incubated with primary antibodies against ABCC5 (1:500, Cell Signaling Technology), caspase-3 (1:1000, Cell Signaling Technology), Bax (1:1000, Abcam), or β-actin (1:5000, Proteintech, Rosemont, USA) and subsequently with IRDye 700 or 800 secondary antibodies (1:10,000, LI-COR Biosciences), and they were visualized using the Odyssey Infrared Imaging System software (LI-COR Biosciences).

### RNA isolation and quantitative real-time PCR (qRT-PCR)

Total RNA was extracted from cells using a RNeasy mini kit (QIAGEN), and qRT-PCR was performed on the cDNAs generated from 250 ng total RNA using the HotStart-IT® SYBR® Green qPCR Master Mix with UDG (2X) and a user-friendly TM kit (USB Corporation). The expression of each gene was calculated using the $2^{{ - \Delta \Delta {\text{C}}_{{\text{T}}} }}$ method with 18S rRNA as an internal control. ABC transporter subfamily primers were designed and synthesized by Sangon Biotech Co., Ltd. (Shanghai, China, Additional file 2: Table S1).

### Immunofluorescence microscopy

Cells were initially seeded onto coverslips, harvested, and washed three times with PBS. Cells were fixed with 4% paraformaldehyde for 30 min at room temperature and blocked with 1% (w/v) bovine serum albumin, 0.1% Triton X-100, and 0.05% Tween-20 at 4 °C overnight to avoid nonspecific staining. Next, the cells were incubated overnight with the goat polyclonal antibody, anti-ABCC5. Subsequently, the rabbit anti-goat secondary antibody (TRITC, 1:100) was added, and the cells were incubated in the dark for 1 h. DAPI staining was used to visualize cell nuclei. Images were captured using a Leica DMI300B inverted fluorescent microscope(Leica Microsystems, Weztlar, Gemany).

### Data analysis

The results are presented as mean ± SD. The graphics and calculations were performed using Microsoft Excel (Microsoft Corp) or Prism 5.0 software. The IC_50_ values were calculated using nonlinear regression from a sigmoidal dose–response curve (variable slope, bottom value 0) using the Prism software. Pearson’s correlation test was used to analyze the correlation between target gene expression and IC_50_ values. A *p* value < 0.05 was considered statistically significant using an unpaired *t*-test unless stated otherwise.

## Results

### Expression of ABC transporters increases in the MCF-7-ADR cell line

The expression of the members of the ABC transporter subfamily was measured using qPCR in resistant MCF-7-ADR and control cells (MCF-7), and 18S RNA expression was utilized as an internal standard to normalize data. Almost all ABC transporters were upregulated in MCF-7-ADR cells as compared to that in control cells. The expression levels of ABCC1 (up to 1.50 times), ABCC2 (1.46 times), ABCC4 (4.31 times), and ABCC5 (5.21 times) were significantly increased, and among them, the change in ABCC5 level was most significant, which indicates its critical role in MTA resistance (Additional file 1: Fig. S2).

### ABCC5 expression in BC tissues correlates with MTA-induced cell toxicity

**Fig. 2:**
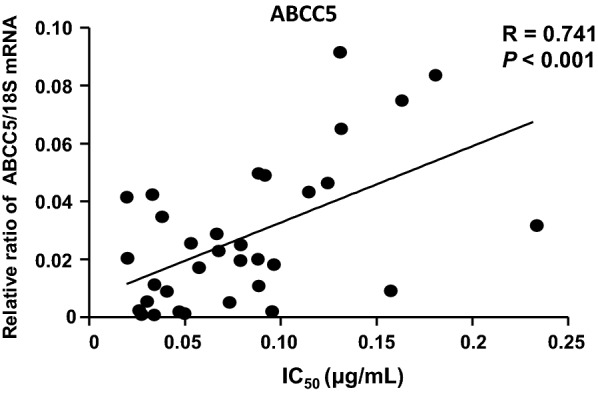
Correlation between the IC_50_ values of MTA and expression of ABCC5 mRNA in primary cell lines from breast cancer (BC) patients (r = 0.741; *p* < 0.001, Pearson’s correlation test). The primary BC cells were cultured with a series of concentrations of MTA for 72 h, and then used to extract RNA for subsequent real-time RT-PCR. The dots represent means of three cell viability assays performed in triplicate, and 18S rRNA was used as an endogenous control

To investigate the correlation between ABCC expression and cellular sensitivity (IC_50_) to MTA in patients, we enrolled 34 BC patients and tested the IC_50_ of primary BC cells isolated from tissue samples. Additionally, we performed mRNA expression analysis of 11 ABC transporter family genes in BC cells (Additional file 1: Fig. S3). Pearson's correlation analysis was performed to evaluate the association between ABCC expression and BC cell viability. Among the ABC transporter subfamilies, the expression of ABCC2 was highest, but no association was observed with MTA sensitivity (R = 0.07, *p* = 0.71). Only ABCC5, as shown in Fig. 2, presented a significant correlation with the IC_50_ of MTA (R = 0.741, *p* < 0.001). ABCC5 may dominate ABC transporter-mediated MTA resistance in BC.

### Effect of ABCC5 on MTA efflux

**Fig. 3:**
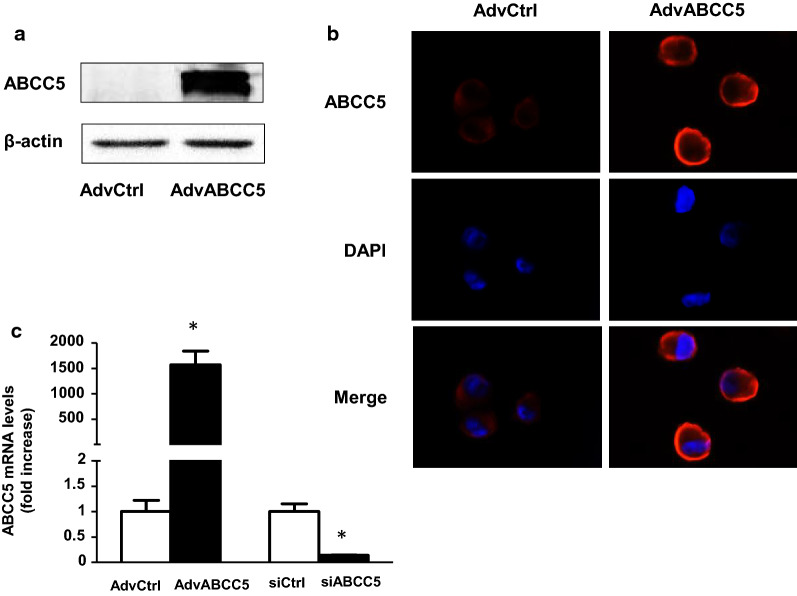
Assessment of transduction of ABCC5 adenovirus in MCF-7 cells. **a** ABCC5 expression was determined using western blotting after transduction with recombinant ABCC5-specific adenoviruses (AdvABCC5; multiplicity of infection (MOI) = 1000) or AdvCtrl (MOI = 1000) in MCF-7 cells for 48 h (n = 4), *p < 0.05 vs AdvCtrl. **b** ABCC5 expression was observed using immunofluorescence microscopy after transduction with recombinant AdvABCC5 or AdvCtrl. **c** ABCC5 mRNA expression determined using RT-PCR after transduction for 48 h. Data represent mean ± SD (n = 3), *p < 0.05

**Fig. 4:**
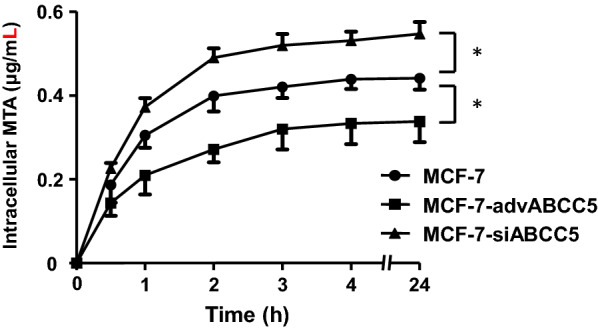
Intracellular concentration–time curve of MTA influenced by the expression of ABCC5 in MCF-7 cells. Data represent mean ± SD (n = 3), *p < 0.05. The cells were incubated at 37 °C with 50 μM (21.37 μg/mL) MTA for 0, 0.5, 1, 2, 3, 4, and 24 h. After washing three times with cold phosphate buffered saline (0.1 M, pH 7.4), the cells were resuspended in RPMI-1640 (0.2 mL) and homogenized. MTA was quantified in the supernatant after centrifugation using RP-HPLC

To validate the functional significance of ABCC5 in drug efflux, we transduced MCF-7 cells with either AdvABCC5 or AdvCtrl. As shown in Fig. 3a, the transduction of MCF-7 cells with AdvABCC5 resulted in a tenfold increase in ABCC5 protein expression. Immunofluorescence microscopy revealed that ABCC5 was expressed on the cell membrane, and an obvious increase in ABCC5 expression was observed in the AdvABCC5 group compared to that in the control (AdvCtrol, Fig. 3b). ABCC5 siRNA was used to knockdown ABCC5 expression, and the results revealed that ABCC5 mRNA expression was completely suppressed (Fig. 3c). Next, we investigated whether alterations in ABCC5 expression in MCF-7 cells could influence the efflux of MTA and thereby alter its intracellular concentration. MCF-7, ABCC5-overexpressing MCF-7, and ABCC5-knockdown MCF-7 cells were treated with 21.37 μg/mL MTA at 37 °C for 0, 0.5, 1, 2, 3, 4, or 24 h. Subsequently, the cells were collected and processed to extract intracellular MTA, and the supernatant was analyzed to determine intracellular MTA concentrations. The intracellular MTA concentration was much lower in ABCC5-overexpressing cells and higher in ABCC5-knockdown cells as compared to that in control cells (*p* < 0.05, Fig. 4). Our results indicate a negative correlation between ABCC5 expression and intracellular MTA concentration in MCF-7 cells.

### Overexpression of ABCC5 weakens the cytotoxicity of MTA

**Fig. 5:**
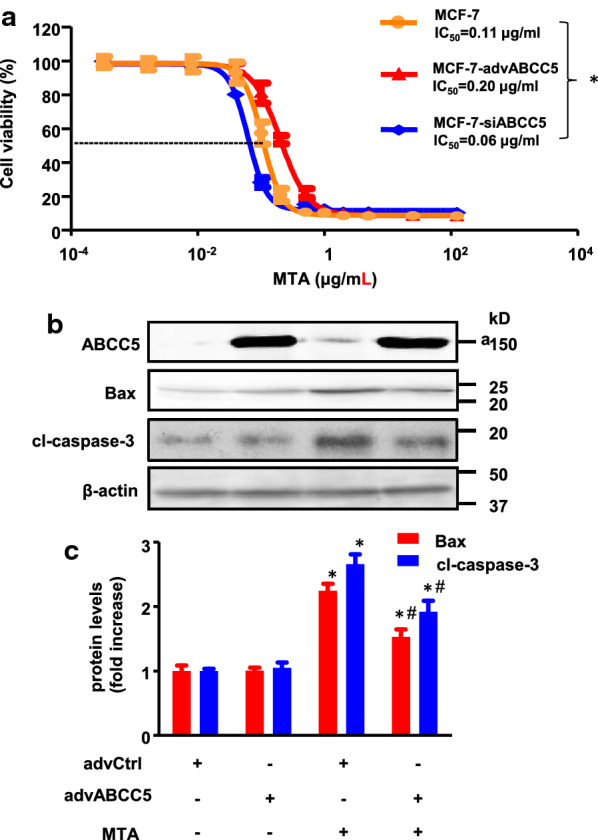
ABCC5 affects the cell viability treated with MTA. **a** Dose–response curves of MCF-7, MCF-7-advABCC5, and MCF-7-siABCC5 cell lines. At least five drug concentrations were used to determine IC_50_ values. Experiments at each concentration were performed in triplicate. The presented data represent the means of three independent experiments. Data are shown as mean ± SD (n = 3, * *p* < 0.05). **b** The transductions of ABCC5 regulate cell apoptosis and expression of Bax and caspase after treatment with 0.1 μM MTA (42.74 ng/mL) for 72 h. The experiments were repeated in triplicate. An equal amount of cell lysate (20 μg of protein/lane) was analyzed using β-actin as the loading control (*p < 0.05, compared with non-MTA treatment group; #*p* < 0.05, compared with AdvCtrl group)

The cytotoxic effect of MTA was evaluated in ABCC5-silenced MCF-7, ABCC5-overexpressing MCF-7, and MCF-7 cells using the CCK-8 assay. As shown in Fig. 5a, the IC_50_ of MTA was significantly decreased after ABCC5 expression was silenced (IC_50_ = 0.06 ± 0.01 and 0.11 ± 0.06 μg/mL for ABCC5-silenced and normal MCF-7 cell lines, respectively, *p* = 0.02), and the IC_50_ increased to 0.2 ± 0.05 μg/mL when ABCC5 expression was upregulated (*p* = 0.003, compared to MCF-7 group). Compared to control cells, an obvious right shift in the dose–response curve was observed in ABCC5-overexpressing cells, and an inverse shift in the curve was observed in ABCC5-knockdown MCF-7 cells. To further investigate MTA-induced apoptosis, the expression levels of cleaved caspase-3 and Bax were measured using western blotting. As shown in Fig. 5b, the expression levels of cleaved caspase-3 and Bax were significantly downregulated (*p* < 0.05) in ABCC5-overexpressing cells, which represents a decline in cell apoptosis.

### ABCC5 affects tumor growth in MTA-treated mice

**Fig. 6:**
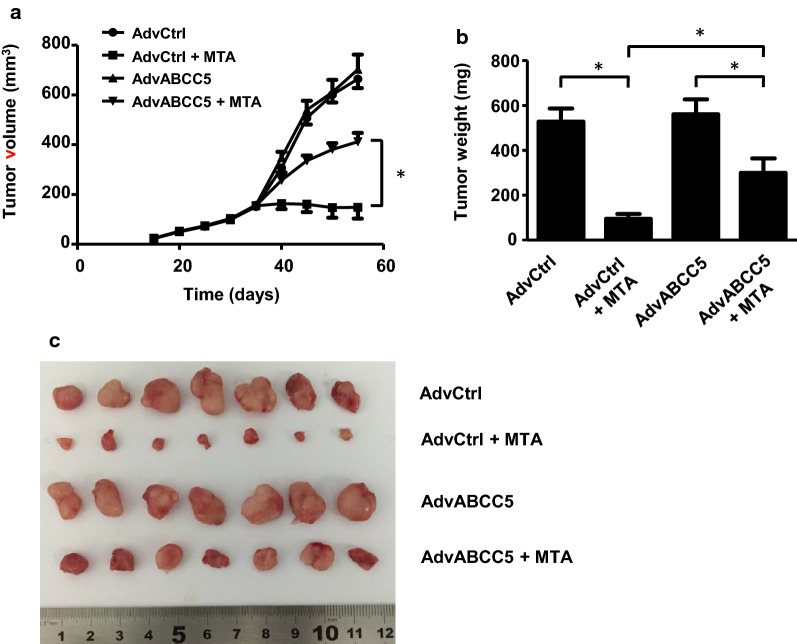
Overexpression of ABCC5 in mice bearing subcutaneous BC xenografts reduces the antitumor activity of MTA. Tumor size was measured with a caliper (**a**). The excised tumors were weighed at the end point (**b**), and data are presented as mean ± SD (n = 6), *p < 0.05. Images of excised tumors from each group are shown (**c**)

The therapeutic effects of MTA were evaluated in mice bearing MCF-7 + AdvCtrl or MCF7 + AdvABCC5 tumor xenografts. As shown in Fig. 6a, MTA treatment (20 mg/kg) resulted in an average 77.7% decrease in tumor volume in mice bearing MCF-7 + AdvCtrl tumor xenografts, whereas it led to a 41.3% decrease in tumor volume in mice bearing MCF7 + AdvABCC5 tumor xenografts. By the end of the experiment, the tumor volume in MTA-untreated mice increased by 4.4-fold than the initial tumor volume (150 mm^3^). The tumor volume in AdvCtrl MTA-treated mice increased by 1.3-fold. whereas the tumor volume in ABCC5-overexpressing MTA-treated mice increased by 2.7-fold (untreated AdvCtrl mice: 665 mm^3^, MTA-treated AdvCtrl mice: 195 mm^3^, untreated AdvABCC5 mice: 703 mm^3^, MTA-treated AdvABCC5 mice: 412 mm^3^; AdvCtrl-MTA group vs. untreated AdvCtrl mice group: *p* < 0.01, AdvABCC5-MTA group vs. untreated AdvABCC5 mice group: *p* < 0.01). Additionally, the tumor size of AdvABCC5 group was significantly higher than that of AdvCtrl group when treated with MTA (*p* < 0.01). These results suggest that ABCC5 overexpression can reduce the cytotoxic effects of MTA in vivo. (Fig. 6b, c).

## Discussion

The expression levels of all multidrug resistant proteins increased in the MCF-7-ADR cell line; in particular, the ABCC5 gene showed the highest increase in expression. This was further confirmed by ABCC5 overexpression in MCF-7 cells, wherein the cells showed a reduced sensitivity, decreased accumulation, and enhanced efflux of MTA, which eventually led to the repression of cell apoptosis. Furthermore, the effect of ABCC5 on MTA resistance was confirmed in an in vivo xenograft model.

Chemotherapy is one of the main options for treating nonresectable BC. However, the development of resistance to chemotherapeutic agents has become a critical problem in clinical practice. Multidrug resistance can be influenced by several factors, and the modulation of expression and function of drug resistant proteins contributes towards resistance [31]. ABC transporters are especially critical for the emergence of multidrug resistance in cancer [32]. It has been reported that the expression levels of many proteins, such as P-glycoprotein, ABC transporters, breast cancer resistance protein (ABCG2), lung resistance-related protein [33], ABCB5 [34], and ABCA8 [35], were upregulated during multidrug resistance development in cell lines, which form a unique defense network against several chemotherapeutic drugs and cellular toxins. Many studies have suggested the role of ABC transporters in the efflux of folate and antifolate drugs [10, 35]. ABCC5 is also involved in the efflux of different anticancer drugs such as 6-MP, 6-TG, 5-FU, and their metabolites [14–19], and this efflux is often associated with drug resistance. Additionally, studies have shown that gene polymorphisms of ABC transporters influence protein expression and determine the efficacy of some anticancer agents [36, 37], despite paradoxical results in some studies.

In our study, we observed that ABCC5 overexpression in MCF-7 cells resulted in an increased resistance to high concentrations of MTA. This can be explained by the increased efflux of MTA from cells via ABCC5, which consequently led to a decrease in MTA accumulation inside the cells, reducing its activity and increasing resistance. In general, it has been observed that upregulated ABCC5 exports nucleoside analogs and increases drug resistance in the range of 2- to tenfold in in vitro assays.

Clinically, the role of ABC transporters in intrinsic or acquired resistance is not clearly understood. Uemura et al. reported that ABCC11 directly confers resistance to MTA by enhancing the efflux of intracellular anti-cancer drugs in lung cancer [38], which suggests that ABCC11 may be a biomarker for MTA in the treatment of lung cancer. Oguri et al. observed that paclitaxel could induce the expression of ABCC10 gene, which then increases paclitaxel resistance by enhancing paclitaxel efflux [39]. In nasopharyngeal carcinoma cells, paclitaxel induces ABCC5 expression through the activation of FOXM1, and its blockage re-sensitizes the cells to paclitaxel [40]. A study has shown that ABCC5 expression is significantly associated with the sensitivities of a panel of non-small-cell lung cancer cell lines to gemcitabine, and inhibition of transporter activity by small molecule inhibitors or siRNA knockdown can significantly resensitize cancer cells to gemcitabine [41]. Moreover, in BC, ABCC5 is significantly overexpressed in the non-responding group after neoadjuvant chemotherapy than in the responding group [42]. Nambaru PK et al. reported that when ABCC5 is upregulated in colorectal cancer and BC, the monophosphorylated metabolite of 5-FU effluxes via ABCC5, which contributes to 5-FU resistance [10]. Other studies have reported that ABCC5 is expressed and functionally active in pancreatic adenocarcinoma cell lines, and it contributes to drug sensitivities [10, 11]. Our study observed a close relationship between ABCC5 expression and cellular sensitivity to MTA in BC cells (R = 0.741), and this phenomenon was accompanied by a significantly decreased accumulation and enhanced efflux of MTA in ABCC5-overexpressing cell lines. In general, a comprehensive profiling of ABC transporters was conducted, which suggested that ABC transporters may serve as promising targets to drastically improve outcomes in cancer treatment.

## Conclusion

Our results showed that the upregulated ABC transporter, ABCC5, was positively correlated with MTA resistance in BC due to an increased efflux of MTA. Thus, ABC transporters may be a new target for anticancer treatment. Further studies are required to quantitatively assess the relationship between ABCC5 expression and MTA dosage in order to evaluate ABCC5 expression level as a biomarker for dose optimization of MTA or new target for BC treatment.

## Supplementary Information

**Additional file 1: Fig. S1.** ABCC5 adenovirus map. **Fig. S2**. Expression of ABC transporters mRNA in MCF-7 and MCF-7ADR cell lines The α-tubulin was used for normalizing of cDNA sample. Results are given as mean ± SD from three separate experiments *p<0.05. **Fig. S3.** Correlation of IC_50_ values of MTA and mRNA expression of ABCC transporters in primary cell lines from patients.

**Additional file 2: Table S1.** The forward and reverse premiers of ABCC transporter sub-family.

## Data Availability

All data and materials are included in the paper.

## Abbreviations

MTA: Pemetrexed
BC: Breast cancer
ABC transporter: ATP-binding cassette transporter
ABCC5: ATP-binding cassette, sub-family C, member 5
RP-HPLC: Reversed phase high performance liquid chromatography
5-FU: 5-Fluorouracil
6-TG: 6-Thioguanine
6-MP: 6-Mercaptopurine
PFU: Plaque formation unit
DMEM: Dulbecco's modified eagle's medium
PBS: Phosphate buffered saline
FBS: Fetal bovine serum
