# Correction to: Human drug efflux transporter ABCC5 confers acquired resistance to pemetrexed in breast cancer

**DOI:** 10.1186/s12935-021-01876-1

**Published:** 2021-03-25

**Authors:** Jihui Chen, Zhipeng Wang, Shouhong Gao, Kejin Wu, Fang Bai, Qiqiang Zhang, Hongyu Wang, Qin Ye, Fengjing Xu, Hong Sun, Yunshu Lu, Yan Liu

**Affiliations:** 1 Department of Pharmacy, Xin Hua Hospital, Shanghai Jiao Tong University, School of Medicine, 1665 Kongjiang Road, Shanghai, 200092 China; 2 Department, of Pharmacy, Changzheng Hospital, Second Military Medical University, Shanghai, 200003 China; 3 Department of Breast Surgery, Obstetrics and Gynecology Hospital, Fudan University, Shanghai, 200011 China; 4 Department of Pharmacy, Provincial Clinical College of Fujian Medical University, Fujian Provincial Hospital, Fuzhou, 350001 China

## Correction to: Cancer Cell Int (2021) 21:136 https://doi.org/10.1186/s12935-021-01842-x

Following publication of the original article [1], we were notified that the email address of Yan Liu should show before the one of Yunshu Lu.

The original article has been corrected.
