# Supplementary material for: Human drug efflux transporter ABCC5 confers acquired resistance to pemetrexed in breast cancer
Source: Cancer Cell Int. 2021 Feb 25;21:136. doi: 10.1186/s12935-021-01842-x (PMC7908708; doi:10.1186/s12935-021-01842-x)
Supplement: Supplementary file 1 — Additional file 1: Fig. S1. ABCC5 adenovirus map. Fig. S2. Expression of ABC transporters mRNA in MCF-7 and MCF-7ADR cell lines The α-tubulin was used for normalizing of cDNA sample. Results are given as mean ± SD from three separate experiments *p<0.05. Fig. S3. Correlation of IC50 values of MTA and mRNA expression of ABCC transporters in primary cell lines from patients. [file 12935_2021_1842_MOESM1_ESM.pptx]

## Slide 1
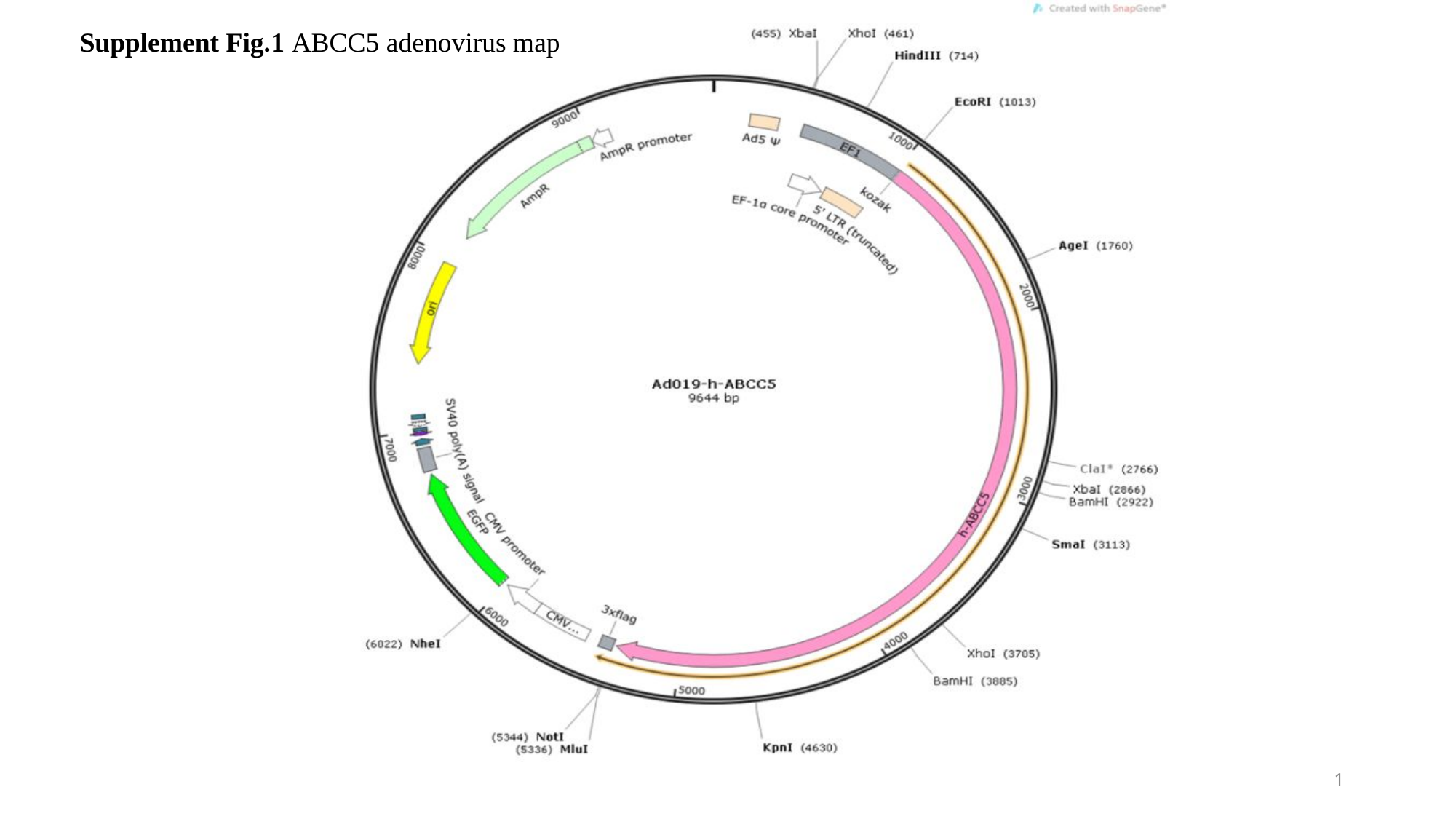

Supplement Fig.1 ABCC5 adenovirus map
1

## Slide 2
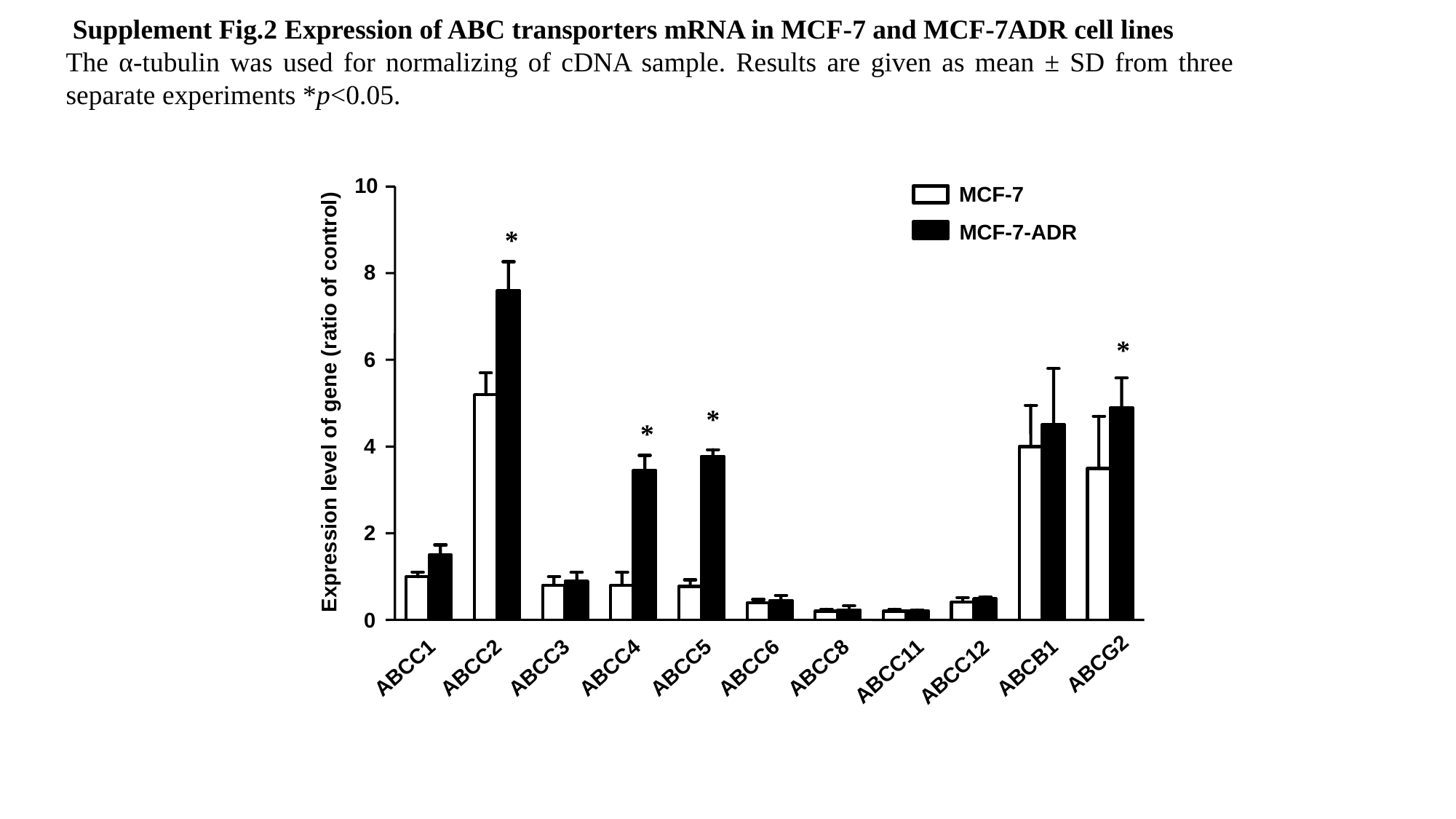

Supplement Fig.2 Expression of ABC transporters mRNA in MCF-7 and MCF-7ADR cell lines
The α-tubulin was used for normalizing of cDNA sample. Results are given as mean ± SD from three separate experiments *p<0.05.
10
MCF-7
MCF-7-ADR
*
8
*
6
Expression level of gene (ratio of control)
*
*
4
2
0
ABCG2
ABCC1
ABCC2
ABCC3
ABCC4
ABCC6
ABCC5
ABCC8
ABCB1
ABCC11
ABCC12

## Slide 3
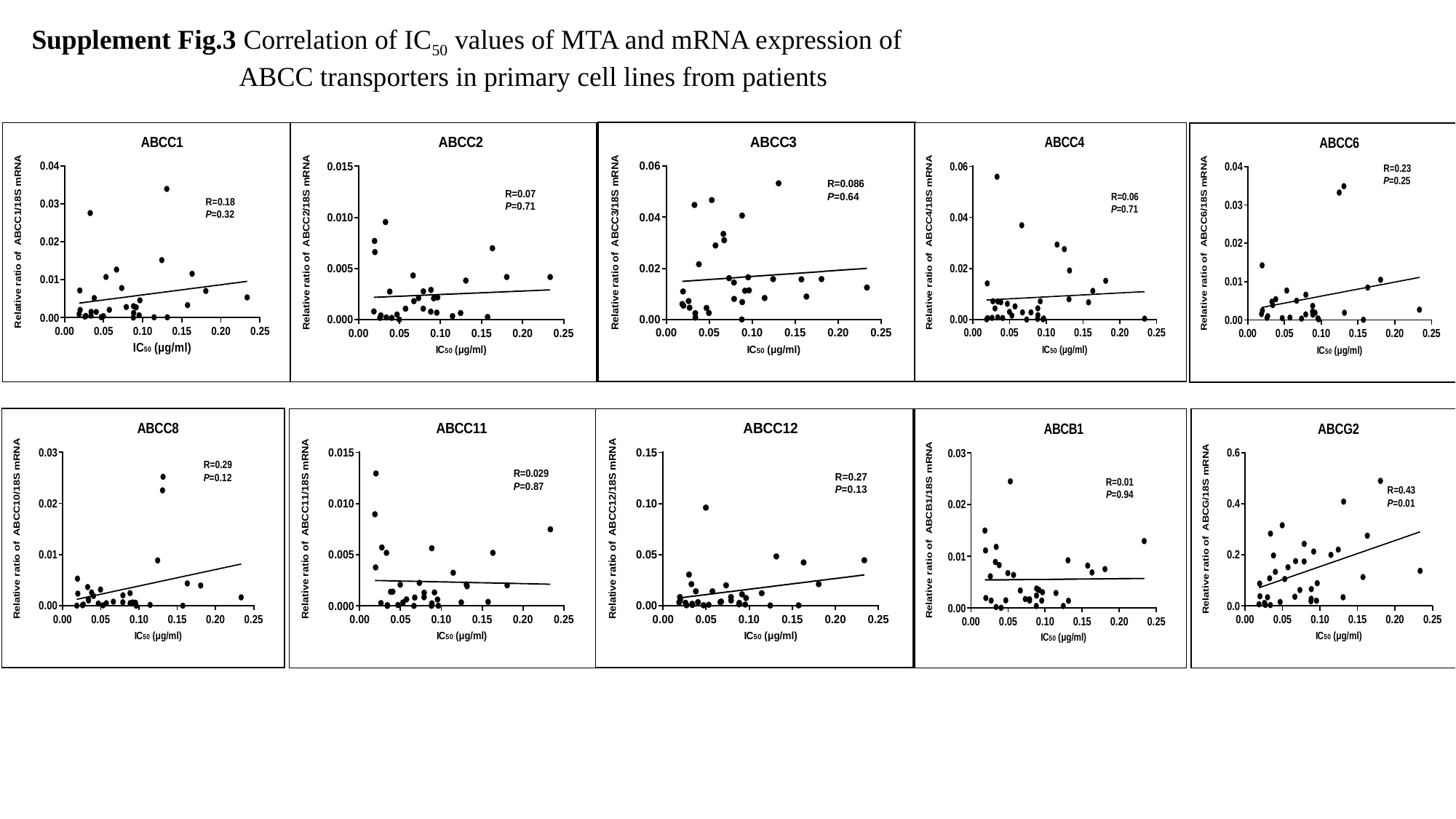

Supplement Fig.3 Correlation of IC50 values of MTA and mRNA expression of
 ABCC transporters in primary cell lines from patients
