# Supplementary material for: Human drug efflux transporter ABCC5 confers acquired resistance to pemetrexed in breast cancer
Source: Cancer Cell Int. 2021 Feb 25;21:136. doi: 10.1186/s12935-021-01842-x (PMC7908708; doi:10.1186/s12935-021-01842-x)
Supplement: Supplementary file 2 — Additional file 2: Table S1. The forward and reverse premiers of ABCC transporter sub-family. [file 12935_2021_1842_MOESM2_ESM.docx]

**Supplementary Table 1**

The forward and reverse premiers of ABCC transporter sub-family

| No. | name | sequence | accession |
| --- | --- | --- | --- |
| 1 | ABCC4 F | GGAGAGCCAAGATACAGAGAATG | NM_005845 |
|  | ABCC4 R | GAGCACCAGCTCTGAAGTAAT |  |
| 2 | ABCC5 F | ACCATCCACGCCTACAATAAA | NM_001023587 |
|  | ABCC5 R | GCATCGCACACGTAAACAAA |  |
| 3 | ABCB1-F | TAATGCGACAGGAGATAGG | NM_000927 |
|  | ABCB1-R | AAGAACAGGACTGATGGC |  |
| 4 | ABCC1-F | GAGGAAGGGAGTTCAGTCTT | NM_019898 |
|  | ABCC1-R | ACAAGACGAGCTGAATGAGT |  |
| 5 | ABCC2-F | CTCACTTCAGCGAGACCG | NM_000392 |
|  | ABCC2-R | CCAGCCAGTTCAGGGTTT |  |
| 6 | ABCC3-F | CTCTTCACTGTGGTCATCCT | NM_003786 |
|  | ABCC3-R | TGGAGATGATGTAGGGGTAG |  |
| 7 | ABCC6-F | GCAACTGGACAGACCTAGAG | U91318 |
|  | ABCC6-R | GAGCTCAGGTCGGTATCTTA |  |
| 8 | ABCC10-F | CGGTTGTCATCTCCATCGTTAT | NM_001198934 |
|  | ABCC10-R | ACCCAAGGGAAGTTGTTGAG |  |
| 9 | ABCC11-F | CTGAGGTTCCAGAGAACAAG | NM_032583 |
|  | ABCC11-R | ACTCAGAGACTTCACACATT |  |
| 10 | ABCC12-F | GGGAACCCACAAGGAGTTAAT | NM_033226 |
|  | ABCC12-R | GCTGCATTGTAAAGGTGTTCAG |  |
| 11 | ABCG2-F | CTCACAATTGCCTACCTGAA | NM_004827 |
|  | ABCG2-R | CTTCAATCAAAGTGCTTCTTTT |  |
